# A Combination of the Kato-Katz Methods and ELISA to Improve the Diagnosis of Clonorchiasis in an Endemic Area, China

**DOI:** 10.1371/journal.pone.0046977

**Published:** 2012-10-08

**Authors:** Su Han, Xiaoli Zhang, Jingshan Wen, Yihong Li, Jing Shu, Hong Ling, Fengmin Zhang

**Affiliations:** 1 Department of Microbiology and Parasitology, The Heilongjiang Key Laboratory of Immunity and Infection, Pathogenic Biology, Harbin Medical University, Harbin, Heilongjiang, China; 2 Key Laboratory of Bio-Pharmaceutical, Harbin Medical University, Ministry of Education, Harbin, Heilongjiang, China; Kenya Medical Research Institute (Kemri), Kenya

## Abstract

**Background:**

Examination of feces by light microscopy is widely used for specific parasitological diagnosis of clonorchiasis. However, the true incidence of infection is underestimated owing to the high missing diagnosis rate of this method. The enzyme-linked immunosorbent assay (ELISA) is widely used for the detection and control of clonorchiasis but the practicality of this method is unclear. The purpose of this study was to evaluate the effect of ELISA as a supplementary method for the diagnosis of clonorchiasis.

**Methodology/Principal Findings:**

The present study recruited 2,359 clinically suspected patients from Heilongjiang Province, China. In all, 954 cases were identified as antibody-positive by immunoglobulin (IgG)-ELISA and 495 individuals were diagnosed as egg-positive by the Kato-Katz (KK) method. The seropositive and egg-negative individuals were re-examined by repeated egg counts and/or the number of KK slides and 18 (18.18%) cases were confirmed as clonorchiasis. The 40.44%, antibody-positive rate determined by IgG-ELISA was significantly higher (*P*<0.05) than the 21.75% egg-positive rate found by examination of feces. A Bayesian approach indicated that the prevalence of clonorchiasis in this region was 22.27% and that the sensitivity, specificity, positive predictive value and negative predictive value of IgG-ELISA were 98.7%, 76.53%, 54.66% and 99.52%, respectively. The agreement between the two methods was moderate (kappa value = 0.564). The clonorchiasis patients lived mainly along the Songhua River. The risk factors, except for ethnic factors, were estimated effectively by both methods.

**Conclusions/Significance:**

The present study suggested that clonorchiasis was widely distributed in Heilongjiang Province, China. The missing diagnosis rate was high using the KK technique alone. The combination of immunological methods and parasitological techniques could improve diagnostic accuracy and reduce the missing diagnosis rate. ELISA used as an auxiliary diagnostic method was realistic and practical for a large-scale screening test, monitoring the prevalence and assessing the risk factors of clonorchiasis.

## Introduction

The oriental or Chinese liver fluke, *Clonorchis sinensis* (*C. sinensis*), is an important food-borne zoonotic parasite. The definitive hosts are infected through consumption of raw freshwater fish containing metacercaria [1]. The adult worms inhabit the intrahepatic bile duct and cause clonorchiasis, which is characterized by hyperplasia and metaplasia in the intrahepatic bile duct epithelium [2]. Persistent and chronic infection often lead to hepatobiliary diseases, including cholangitis, cholelithiasis, cholecystitis, pancreatitis, hepatic fibrosis, cholangiocarcinoma and liver cancer [3], [4], [5]. *C. sinensis* has been classified by the International Agency for Research on Cancer (IARC) as a group 1 biocarcinogen to humans [6]. It is estimated that nearly 601 million people are at risk and 35 million people are infected worldwide [7], [8]. *C. sinensis* is endemic predominantly in Asia, particularly China, Japan, Korea and Vietnam [9]. In China, approximately 15 million people are estimated to be infected, mainly in the southeast and northeast areas, such as Guangdong, Guangxi and Heilongjiang Provinces [8]. According to national sampling surveys, the prevalence of clonorchiasis as a representative of food-borne parasitical disease increased from 1990–2003 [9], [10]. Furthermore, clonorchiasis is the subject of national parasitic disease control programs in China with a projected timescale of 2006–2015.

Currently, fecal examination is mainly used for specific parasitological diagnosis of clonorchiasis. The Kato-Katz (KK) technique, a diagnostic method used extensively, is easy to operate, rapid and cheap, and can be used to estimate the intensity of infection [11]. However, the sensitivity of the KK technique is low, especially for low levels of infection intensity [12]. The serum enzyme-linked immunosorbent assay (ELISA) is sensitive, inexpensive and easily repeated, making it superior to parasitological examinations [13]; however, owing to a low level of specificity, it is easy to obtain false-positive results [14]. In addition, the practicality of using ELISA as supplementary diagnosis remains unclear, particularly in clonorchiasis endemic regions.

Recently, Bayesian statistical methods are increasingly used in the analysis of parasitological data [15]. Bayesian analysis has been used for estimating measures of test accuracy and prevalence inference in epidemiological studies of many parasitic diseases, such as schistosomiasis, onchocerciasis and giardiasis [16], [17], [18], [19]. The Bayesian model, which could evaluate the sensitivity and specificity of various tests in the absence of a gold standard, can help estimate the most suitable test for routine surveillance and diagnosis [20], [21]. Therefore, the Bayesian method was used to estimate the test properties of the KK and ELISA methods in the present study.

This study compared the results of using the KK and ELISA methods in clinically suspected patients referred to the Department of Parasitology, Harbin Medical University in Heilongjiang Province for diagnosis. The purpose of this study was to evaluate the effect of ELISA as a supplementary method for the diagnosis of clonorchiasis, aiming to improve the diagnostic accuracy and to provide measures for the prevention and control of clonorchiasis.

## Materials and Methods

### Location and Study Participants

Heilongjiang Province, with Harbin City as its capital, is located in the northeast of mainland China, and shares borders with Jilin Province to the south and with Russia to the northeast. It has a continental warm climate with rainy and hot summers (the rainy season is between May and October) and with cold and snowy winters. There are several fishponds and rivers, including the Heilong jiang (River), the Songhua River, the Ussuri River and the Nen River. The province is divided into 13 administrative regions and, of the 38 million people who live in this area, most are Han Chinese but there are several ethnic minorities, including Manchus, Koreans and Mongols [22].

According to the national standardized diagnostic criteria for suspected patients published by the Ministry of Health of China (WS309-2009), a total of 2359 clinically suspected clonorchiasis patients (639 females and 1720 males; age 5–86 years) were sampled and referred to the parasite institute of Harbin Medical University in Heilongjiang Province from 2009 to 2011. They had developed clinical symptoms and signs such as discomfort, anorexia, indigestion, abdominal pain, abnormal liver function and rising eosinophilia (>500–1000/µl of blood). They had been living in, or had travelled to, an endemic region where ingestion of raw freshwater fish was common.

### Examination of Feces

Stool samples were collected from each participant and inspected for the presence of *C. sinensis* eggs by the KK method. Briefly, each fecal sample was sieved through a screen, placed into a hole in a plastic template (41.7 mg) on a glass slide, covered with cellophane soaked in glycerol and malachite green, then pressed against a hard surface to spread the sample evenly. After clarification overnight, triplicate slides were made for each specimen. At 1–12 h after preparation, the slides were examined for *C. sinensis* eggs under a light microscope by two experienced technicians who were blinded to the participant’s medical status [12], [23].

### Diagnosis of Blood Samples

Serum specimens prepared from peripheral blood samples of participants were tested to quantify the level of *C. sinensis* antigen-specific IgG antibody using an indirect ELISA diagnostic kit (Shenzhen Combined Biotech Co.Ltd., China). All washing and detection steps were done following the manufacturer’s instructions. All serum samples were diluted 1∶100 in sample diluent, transferred into the kit’s microtiter wells and measured with a micro plate reader at 492 nm. The optical density (OD) values of all serum greater than 2.1 times of mean OD value of the negative control serum were defined as antibody-positive according to the manufacturer’s instruction, while the OD values of samples less than 2.1 times of mean OD value of the negative control serum were regarded as antibody-negative.

### Re-examination of Feces

We randomly sampled 200 seropositive and egg-negative individuals and performed re-examination by repeated egg counts and/or the number of KK slides. However, because of financial, distances, time or other reasons, re-examination could not be conducted on some cases. In addition, some cases believe that clonorchiasis is not important, thus re-examination is neglected and even forgotten.

### Questionnaire Investigation

All participants were interviewed regarding life-style habits and related dates to gather information on gender, ethnicity, residence, treatment experience and history of eating raw freshwater fish and/or shellfish.

### Ethics Statement

The procedures of sample collection and use were in accordance with permission granted by the Human Ethics Review Board, Harbin Medical University. The objectives, procedures and potential risks were explained to all participants. Written informed consent was obtained directly from all adult participants. If the participants were children, written informed consent were obtained from the next of kin, carers or guardians on the behalf of the minors/children participants. Individuals with positive fecal examination results were treated with a 25 mg/kg oral dose of praziquantel three times a day.

### Statistical Analysis

Statistical analysis was done with SPSS software (version 10.0 for Windows; Chicago, IL, USA). The χ^2^ test was used to assess the association between qualitative variables. Unconditional multiple logistic regression was used to calculate odds ratios (ORs) and corresponding 95% confidence intervals (CIs) of being *C. sinensis* egg-positive according to various characteristics. *P*≤0.05 was set as the level of statistically significant difference.

The agreement of inter-specimen readings was assessed categorically, using kappa statistics. We used the following guidelines to interpret the kappa values: <0.2, poor agreement; 0.2–0.4; fair agreement; 0.4–06, moderate agreement; 0.6–0.8, good agreement; and 0.8–1.0, excellent agreement.

The sensitivity and specificity of the two diagnostic tests (ELISA and KK) and the prevalence of clonorchiasis were estimated in the absence of a gold standard using the Bayesian method [24]. Prior information about the sensitivity and specificity and prevalence of infection were quantified using beta (α, β) distributions. The prior information was based on previously published reports and presented in Table 1. The freeware program WinBUGS 1.4 was used to run all models using Gibbs sampling. A Markov chain Monte Carlo simulation was conducted to estimate the mean and 95% probability intervals (credibility intervals) for all parameters from the respective posterior distributions. The Markov chain was run for 32000 iterations with a burn-in of 2000 iterations. We discarded an initial burn-in of 2,000 iterations and ran for another 30000 iterations to obtain estimates.

**Table 1:** Values of priors and corresponding beta distributions used to estimate the test parameters in the diagnosis of *Clonorchis sinensis*.

	Parameter	95% probability ranges (%)	Beta (α, β) priordistribution	Source of priorprobabilities
Kato-Katz	Se	0.90–1.00	71.25, 3.75	Ref [12], [39]
	Sp	0.75–1.00	23.625, 3.375	Ref [12], [39]
ELISA	Se	0.50–1.00	8.25, 2.75	Ref [14], [40]
	Sp	0.00–1.00	1,1	Ref [14], [40]

Se = sensitivity.

Sp = specificity.

## Results

### Parasitological and Immunological Examinations

**Figure 1:**
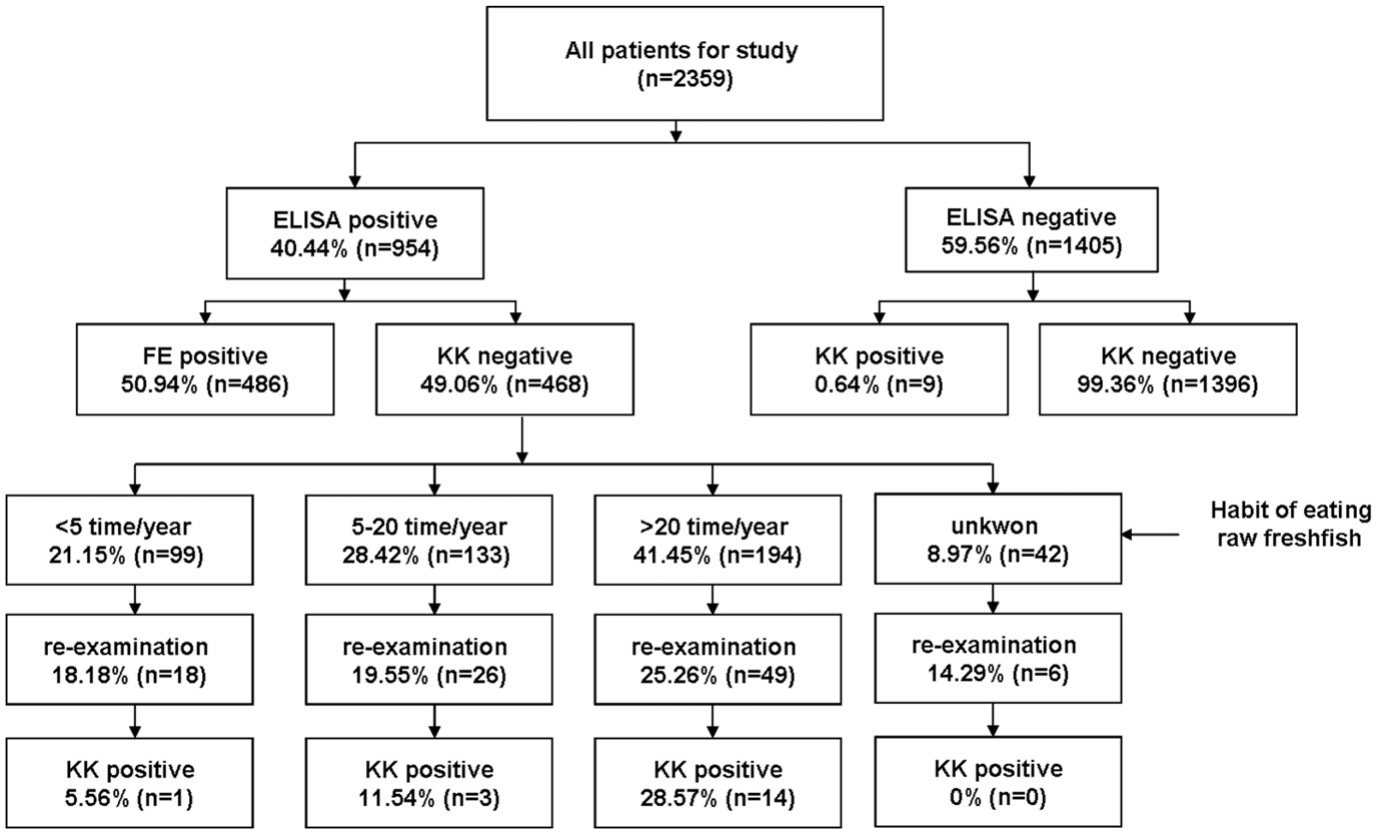
Flowchart for the diagnosis of 2359 patients. KK = Kato-Katz technique.

IgG-ELISA was used as a first screening step, followed by the KK technique to confirm infection based on the presence of *C. sinensis* eggs in stools. Fig. 1 shows the experimental design and the prevalence of *C. sinensis* observed by the two methods. We found 954 seropositive and 1405 seronegative participants using IgG-ELISA. In the group of seronegative individuals, 9 were egg-positive and 1396 were egg-negative as determined by the KK method. In the seropositive cases, 486 cases were egg-positive and 468 were egg-negative. In the random sample of 200 seropositive and egg-negative individuals, 99 cases were subsequently subjected to re-examination. Through statistical analysis, the 99 cases could represent the characteristics of 200 cases. Of 99 cases, clonorchiasis by repeated egg counts and/or the number of KK slides were identified in 18 (18.18%) cases. The majority of the egg-positive cases (77.78%) had consumed raw freshwater fish >20 times per year.

The total number of egg-positive specimens was 513; the overall prevalence of *C. sinensis* infection among the study participants was 40.44% (954/2359) and 21.75% (513/2359), as determined by the IgG-ELISA and KK methods, respectively (Table 2). Statistical analysis showed the kappa value was 0.564 between the two methods and the agreement was moderate.

**Table 2:** Comparison of the Kato-Katz methods and ELISA for diagnosis *Clonorchis sinensis* infection in Heilongjiang Province.

	ELISA		
Kato-Katz	No.positive	No.negative	Total
No.positive	504	9	513
No.negative	450	1396	1846
Total	954	1405	2359

The Kappa value between the two methods was 0.564, *P*<0.001.

The test properties of the KK and IgG-ELISA methods, determined by the Bayesian analysis, are given in Table 3. Sensitivity, specificity, positive predictive value (PPV) and negative predictive value (NPV) of IgG-ELISA were 98.7%, 76.53%, 54.66% and 99.52%, respectively.

**Table 3:** Test properties of the Kato-Katz methods and ELISA as estimated by Bayesian analysis.

Parameter	Kato-Katz	ELISA
	Total % (95% CI*)	Total % (95% CI)
Sensitivity	95.4(90.34–98.75)	98.7(97.23–99.7)
Specificity	99.41(98.84–99.82)	76.53(74.33–78.78)
Positive predictive value	97.89(95.87–99.36)	54.66(51.01–58.69)
Negative predictive value	98.68(97.12–99.65)	99.52(98.96–99.89)

* CI, credibility intervals.

### Prevalence of *C. sinensis* Near River Basins

**Figure 2:**
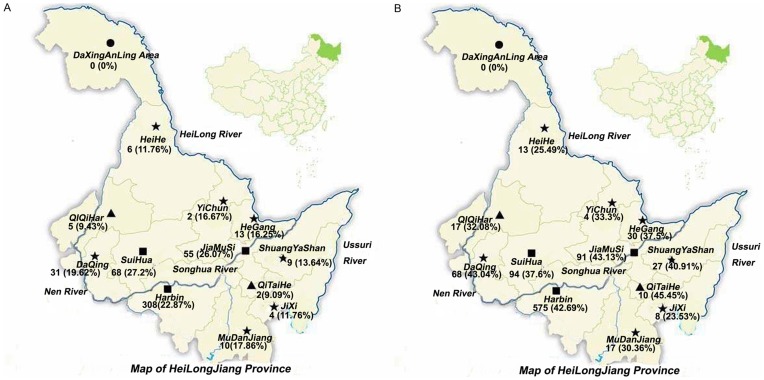
Regional distribution of *Clonorchis sinensis* infection in Heilongjiang Province by Kato-Katz and ELISA examinations. A. by Kato-Katz B. by ELISA.

The prevalence of *C. sinensis* near rivers and the surrounding areas was determined using the KK and IgG-ELISA methods (Table 4; Fig. 2). All of the suspected patients in this study came from 13 administrative regions in Heilongjiang Province, mainly from the Harbin (57.1%), Suihua (10.6%) and Jiamusi (8.94%) areas.

**Table 4:** Regional distribution of *Clonorchis sinensis* infection in Heilongjiang Province by Kato-Katz and ELISA examinations.

Riversides	Locality	No. examined	No. positive (%)	
			Kato-Katz	ELISA
*Songhua River*	*SuiHua*	250	68(27.2)	94(37.6)
	*Jiamusi*	211	55(26.07)	91(43.13)
	*Harbin*	1347	308(22.87)	575(42.69)
	*MuDanJiang*	56	10(17.86)	17(30.36)
	*QiTaiHe*	22	2(9.09)	10(45.45)
	*Subtotal*	1886	443(23.49)	787(41.73)
*Nen River*	*DaQing*	158	31(19.62)	68(43.04)
	*QiQiHar*	53	5(9.43)	17(32.08)
	*Subtotal*	211	36(17.06)	85(40.28)
*Ussuri River*	*ShuangYaShan*	66	9(13.64)	27(40.91)
	*JiXi*	34	4(11.76)	8(23.53)
	*Subtotal*	100	13(13)	35(35)
*Heilong River*	*HeGang*	80	13(16.25)	30(37.5)
	*YiChun*	12	2(16.67)	4(33.3)
	*HeiHe*	51	6(11.76)	13(25.49)
	*DaXingAnLing Area*	19	0(0)	0(0)
	*Subtotal*	162	21(12.96)	47(29.01)
Total		2359	513(21.75)	954(40.44)

Except for the Daxinganling area, the *C. sinensis*-infected cases were found by both methods in 12 administrative regions. The highest egg positive rate (23.49%) was found in the Songhua River basin (from 9.09 to 27.2% in different counties). The prevalence of *C. sinensis* was 27.2%, 26.07% and 22.87% in three townships along the Songhua River: SuiHua, Jiamusi and Harbin, respectively. In the Nen River basin, the average egg-positive rate was 17.06% (range 9.43–19.62%). The egg-positive rate of *C. sinensis* was 13% and 12.96% in the Ussuri and Heilong River basins, respectively. The results of the IgG-ELISA method showed the same prevalence tendency by locality.

### Epidemiological Factors of *C. sinensis*

Characteristic risk factors were evaluated by comparing the results obtained by the two methods (Table 5). Males showed a significantly higher (*P*<0.001) egg-positive rate (24.19%) than females (15.18%). Males were at greater risk than females (OR in males *versus* females = 1.783; 95% CI, 1.399–2.272). The antibody-positive rate determined by IgG-ELISA was 1.29-fold greater (*P*<0.001) in males (43.08%) than in females (33.33%).

**Table 5:** Characteristics of Human *Clonorchis sinensis* infection in Heilongjiang Province detected by Kato-Katz and ELISA examinations.

Variable	Subcategory	No. examined	No. positive (%)			No. negative (%)
			Kato-Katz	ELISA	Kato-Katzand ELISA	Kato-Katzand ELISA
Total		2359	513(21.75)	954(40.44)	504(21.36)	1396(59.18)
Gender	Male	1720	416(24.19)*	741(43.08)*	409(23.78)*	972(56.51)*
	Female	639	97(15.18)	213(33.33)	95(14.87)	424(66.35)
Age (years)	<29	225	28(12.44)*	62(27.68)*	28(21.21)*	161(71.56)*
	>29	2134	485(22.73)	892(41.78)	476(37.93)	1235(98.41)
Race	Korean	53	18(33.96)*	21(39.62)	17(32.08)	31(58.49)
	Others	2306	495(21.47)	933(40.46)	487(21.12)	1365(59.19)
Religion	Cities	629	78(12.40)*	185(29.41)*	78(12.40)*	444(70.59)*
	Countrysides	1730	435(25.14)	769(44.45)	426(24.62)	952(55.03)
Treatment experience	Yes	201	46(22.88)	187(93.03)*	45(8.92)	12(0.86)*
	No	2158	467(21.64)	767(35.54)	459(91.07)	1384(99.14)

* *P*<0.05.

**Figure 3:**
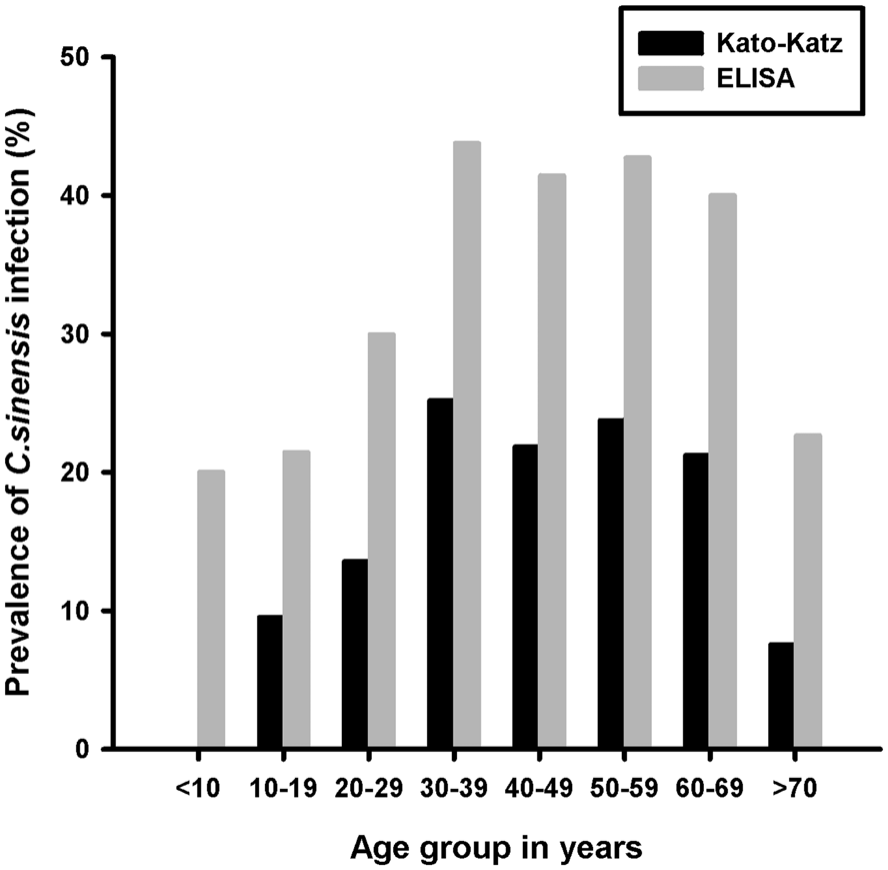
Prevalence of Clonorchis sinensis infection in different age group by Kato-Katz and ELISA examinations.

With regard to age, KK examination found no subject aged <17 years or >81 years infected with *C. sinensi*s. The prevalence increased to a rather constant high level in participants aged 20–69 years, reached a peak at ages 30–39 years and declined to a lower rate at ages >39 years. However, one egg-negative child was seropositive and 22.64% of seropositive cases were aged >70 years. The seroprevalence of clonorchiasis detected by IgG-ELISA was 1.8–3 fold greater than the prevalence determined by the KK method (Fig. 3).

According to the KK method, the majority (33.96%) of infected people in this study were Korean, significantly greater (*P*<0.05) than the proportion of any other nationality (21.47%). However, the antibody-positive rate in Koreans was low. The rate of infection in rural areas was higher than that found in the city by both methods and the re-infection rate was 22.88% and 93.03% as judged by the KK and the ELISA techniques, respectively. In general, with the exception of ethnic factors, similar prevalence tendencies were estimated using ELISA alone or both KK and ELISA.

## Discussion

Clonorchiasis has been identified as a public health problem in Heilongjiang Province [22]. Because of the changing epidemiological pattern and eating habits, as well as the rapid growth of aquaculture and food distribution networks, the infection rate of *C. sinensis* could still be increasing [10], [25]. Clonorchiasis presenting without special clinical symptoms is among the most neglected tropical diseases [26]. At present, oral dosage with praziquantel is the most common approach for the treatment and control of clonorchiasis [27]. Identification of target individuals or communities for chemotherapy, indirect evaluation of morbidity and application of control strategies all depend on accurate results of diagnostic tests [5], [22]. Because re-infection is very common, the test-treat approach is probably the most cost-effective way to meet the needs for repeated re-treatments [15]. Therefore, accurate and rapid diagnosis of clonorchiasis appears to be of the utmost importance.

Post-mortem examination and to an extent parasite expulsion is the only gold standard to date for diagnosis of intestinal parasites. However, these methods are impractical to carry out as part of the routine surveillance and diagnostic tests. In the absence of a gold standard, Bayesian statistical method was employed to estimate the parameters of the diagnostic tests applied in this study. To the best of our knowledge, this is the first published application using a Bayesian approach to evaluate the diagnostic tests for clonorchiasis. In the present study, the prevalence of *C. sinensis* estimated by Bayesian analysis was 22.27%. Wang et al. made a survey on 282 subjects suspected of clonorchiasis and reported the prevalence was 21.63% [28]. In another survey during 1993–2006, the prevalence was from 1.6% to 9.0% for Korean patients suspected of having parasitic infection [29]. The difference could be related to factors such as experimental design, sensitivity and specificity of the detection methods, and overall sample size.

Because the eggs are similar in size and are all oval and operculated, *C. sinensis* eggs cannot be differentiated from other Heterophyidae (e.g. *Haplorchis*, *Heterophyes* etc.) or Opisthorchiidae eggs [30]. However, in China, no *Opisthorchis felineus* infection has been reported and *Opisthorchis viverrini* infection has been reported only in Taiwan [31]. Human infections with other minute intestinal flukes have been found sporadically in several areas, such as Guangdong Province, Taiwan and Guangxi Zhuang Autonomous Region(GZAR) [32], [33]. Recently, Min HC et al. surveyed the prevalence of *C. sinensis* in Heilongjiang Province from 2001 to 2004 and did not find other intestinal trematode and minute intestinal flukes infections [22]. In summary, there is no evidence that other fish-borne trematode infections with similar egg morphology such as minute intestinal flukes are endemic in Heilongjiang Province. Therefore, there is no difficulty in differential diagnosis between *C.sinensis* and other minute intestinal flukes. However, its high missing rate and insensitivity lead to false-negative results, limiting its reliability. There are many other reasons that could explain why the KK method has a high missing diagnosis rate. First, because few eggs are produced with little day-to-day variation, the egg count in feces often fluctuates dynamically. Second, *C. sinensis* eggs are the smallest eggs in helminth-parasitized humans and are often covered by the fecal matter. Finally, it is not feasible to detect the eggs in patients with biliary obstruction entirely or in case of low infection intensity. Although the number of false-negative cases is reduced by increasing the size of the stool sample, repeated egg counts and/or the number of KK slides [12], some infections are missed by the KK technique. Moreover, repeated collection and examination of stools is time-consuming, labor-intensive and might not be acceptable to communities. Therefore, it is imperative to improve the utility of diagnosis of *C. sinensis* and reduce the missing rate.

Owing to high sensitivity and low specificity, the ELISA method is commonly used in the diagnosis of *C. sinensis*. This finding demonstrated that the antibody-positive rate determined by ELISA was 40.44%, significantly higher (*P*<0.05) than the egg-positive rate obtained by the KK approach (21.75%). The kappa value between the two methods was 0.564.

A total of 450 seropositive cases were identified in the egg-negative individuals. The specificity of the IgG-ELISA method was fairly poor (76.53%). One possible explanation for this observation is that because *cysticercosis* and *Taeniasis solium* are endemic in this region, it is likely to produce the cross-reactions with these parasites [10], [34]. Another reason is the slow decrease of the antibodies after treatment, which produced false-positive results [35].

Of 99 cases subjected to re-examination, 18 were identified as clonorchiasis by repeated egg counts and/or the number of KK slides. We proposed that these patients were missed easily by the KK method because they were probably infected in the early or late phase (with a greater bile duct obstruction). As a result, we recommended that selective repetitive examinations of stools might be applied in seropositive and egg-negative individuals for definite diagnosis, which could reduce the rate of misdiagnosis and improve the efficiency of diagnosis.

Additionally, 9 patients were seronegative and egg-positive. A possible interpretation for this poor performance of ELISA in the detection of *C. sinensis* was that these patients might have had low antibody responses. Secondly, it is suggested that the levels of IgG antibody increased to above that of the controls only as the numbers of worms increased [35]. Thirdly, it needs at least 2 weeks for IgG antibody to become positive in the early phase of moderate or heavy infection by *C. sinensis* [35]. Some cases were re-infected with *C. sinensis* in the endemic area after their IgG antibody had disappeared.

The NPV of IgG-ELISA was excellent (99.52%), which implied that individuals are detected as seronegative first with ELISA and no further estimation or treatment was undertaken. The way to deal with parasitic infections that have unclear symptomatology and for which there are safe, efficacious and low-cost drugs is by treating at regular intervals in communities where the infection is intensively transmitted. This strategy is presently used for the control of many parasites, such as schistosomiasis, *lymphatic filariasis* and onchocerciasis [36]. The ELISA method is valuable for identification of communities in need of large-scale administration of Praziquantel. Furthermore, the ELISA method could represent an essential tool to conduct community diagnosis followed by mass drug distribution accompanied by health education campaigns aiming at behavioral change. However, the ELISA method was not used instead of KK technology. Hence, targeting individuals with chemotherapy should not rely on the results of the ELISA method alone, it would be better to supplement it with examination of stools.

With more population migration and change of food culture, monitoring the change of risk factors is important for control of clonorchiasis. Except for the ethnic factors, the risk factors were evaluated effectively by the two methods. Our results suggested that a single ELISA method could have been used for a large-scale screening test to monitor the prevalence and assess the risk factors. Further, this single method will reduce the detection personnel workload and avoid the need for stool examination and thus harbor human resources.

Recently, molecular biological methods such as the nested PCR, multiplex PCR, real time PCR and LAMP have been developed for diagnosis of *C. sinensis* in human and animals [37], [38], [39]. However, these methods are too complicated and inconvenient for large-scale epidemiological investigation, owing to the need for laboratory facilities, trained personnel and more financial resources. These molecular methods seem to be impractical for point-of-care diagnosis of *C. sinensis*. Although PCR/LAMP methods are limited by their high cost and need for special labs, they could be used as a research tool for primary evaluation of diagnostic test parameters. The ELISA method is a relatively satisfactory diagnostic method due to its sensitivity, inexpensiveness and easy repetition, which has advantages for specific situations. While, the ELISA method has limitations and still needs further improvement in the future so as to diagnose *C. sinensis* more rapidly and effectively. In addition, the sensitivity and specificity of the current ELISA could be validated by the molecular biological methods in future. So, we propose a combination of immunological methods (ELISA) and parasitological technology (KK) to improve diagnostic accuracy and reduce the missing diagnosis rate, especially in individuals with mild symptoms.

This study has some limitations. First, because a considerable number of seropositive individuals might be missed without re-examination, the prevalence of clonorchiasis was underestimated to some degree. In addition, we repeatedly collected stool samples only in the seropositive cases, but those with both KK and ELISA negative were not investigated further.

In conclusion, the results of the present study suggest that suspected hepatobiliary patients should be examined for clonorchiasis in endemic areas. Each diagnostic method has advantages for specific situations. We believe that a proper evaluation of any epidemic situation requires the combination of immunological methods and parasitological technology. We advised seropositive, egg negative patients to be re-examined. ELISA, as an auxiliary diagnostic method, was applicable and valuable for large-scale screening tests, monitoring the prevalence as well as assessing risk factors of clonorchiasis.
